# Effects of l‐carnitine on frailty status in patients with liver cirrhosis: A randomized‐controlled trial

**DOI:** 10.1002/hsr2.70148

**Published:** 2024-10-27

**Authors:** Nasrin Motazedian, Anita Ashari, Niloofar Dehdari Ebrahimi, Mehrab Sayadi, Sarina Pourjafar, Nazanin Motazedian, Vahid Khademi, Alireza Shamsaeefar, Ahad Eshraghian

**Affiliations:** ^1^ Transplant Research Center Shiraz University of Medical Sciences Shiraz Iran; ^2^ Shiraz Geriatric Research Center Shiraz University of Medical Sciences Shiraz Iran; ^3^ Cardiovascular Research Center Shiraz University of Medical Sciences Shiraz Iran; ^4^ Student Research Committee Jahrom University of Medical Sciences Jahrom Iran; ^5^ Organ Transplant Center, Shiraz University of Medical Sciences Shiraz Iran

**Keywords:** carnitine, frailty, liver cirrhosis, liver function

## Abstract

**Background and Aims:**

Frailty is a common complication in patients with liver cirrhosis, which is linked with augmented rates of morbidity and mortality. In this regard, timely nutritional assessment and intervention have gained scientific attention. L‐carnitine may be a promising candidate with its potential to enhance energy metabolism, reduce inflammation, and act as an antioxidant. Therefore, we aimed to assess the impact of l‐carnitine supplementation on frailty status and liver function in adults with liver cirrhosis.

**Methods:**

This double‐blinded, randomized, controlled clinical trial study enrolled 77 patients with liver cirrhosis. Patients were randomly allocated into two groups: the control group (*n* = 42) and the l‐carnitine group (*n* = 35). The l‐carnitine group received 500 mg of l‐carnitine orally three times a day for 8 weeks, while the control group did not receive any intervention.

**Results:**

L‐carnitine administration resulted in a significant decrease in alanine transaminase levels (*p*: 0.043) and partial thromboplastin time (*p*: 0.036). Furthermore, compared to the control group, l‐carnitine treatment led to improvements in prothrombin time (*p*: 0.008) and international normalized ratio (*p*: 0.024). However, no significant improvement in the Liver Frailty Index, Freid Frailty Index, and Karnofsky Performance Status Scale (*p* > 0.05) was observed in the carnitine group after the 8‐week intervention period.

**Conclusion:**

In conclusion, the administration of l‐carnitine exhibited hepatoprotective properties and was correlated with lowered alanine transaminase levels with improvement in coagulation status in liver cirrhosis patients. Nevertheless, our study indicated that the short‐term use of l‐carnitine might not significantly improve frailty in these patients.

## 1 INTRODUCTION

Frailty is a complex clinical condition characterized by aging‐associated perturbation in various physiological systems that results in enhanced susceptibility to stressors and decreased physiological reserves and function. The main components of frailty are sarcopenia and physical impairment, resulting in reduced physiological capacity over time. In most cases, frailty can be caused secondary to a chronic disease such as malignancies, inflammatory diseases, and chronic liver disease.^1^, ^2^ Sarcopenia is highly prevalent in patients diagnosed with liver cirrhosis, affecting around 14%–78% of such patients.^3^ Recent studies have indicated that frailty and sarcopenia are associated with higher morbidity and mortality rates in individuals with liver cirrhosis.^4^, ^5^

The pathophysiology of frailty and sarcopenia in liver cirrhosis is multifactorial, involving alterations in protein metabolism, increased oxidative stress, and systemic inflammation. Liver impairment significantly disrupts the metabolism of lipids, proteins, and carbohydrates. Notably, alterations in protein metabolism, especially branched‐chain amino acids, which are essential for detoxifying extrahepatic ammonia, lead to decreased circulating levels of these amino acids. This reduction can precipitate an accelerated rate of muscle breakdown. Additionally, compromised hepatic function and increased portosystemic shunting elevate systemic ammonia concentrations, further impairing muscle integrity.^6^ In this regard, antioxidants have gained scientific interest due their potential role in mitigating frailty among patients with liver cirrhosis, one of these agents is l‐carnitine.

L‐carnitine (β‐hydroxy‐γ‐N‐trimethyl aminobutyric acid) is an amino acid derivative that plays a critical role in energy metabolism by facilitating fatty acid transportation into the mitochondria for β‐oxidation.^7^ Carnitine deficiency can prohibit vital metabolic processes in the liver, including albumin biosynthesis, fatty acid metabolism, ammonia reduction by the urea cycle, and gluconeogenesis. These metabolic derangements can result in hyperammonemia and hypoalbuminemia, leading to several complications associated with liver cirrhosis such as muscle cramps, hepatic encephalopathy. Thus, l‐carnitine emerges as a potential therapeutic agent for ameliorating sarcopenia.^8^

Recent studies have demonstrated that l‐carnitine administration may mitigate skeletal muscle wasting, prevent sarcopenia progression, and improve neurological function in patients with liver cirrhosis.^9^, ^10^ Furthermore, l‐carnitine supplementation has shown protective effects against skeletal muscle depletion, even in the absence of decreased ammonia levels, possibly due to its antioxidant and anti‐inflammatory properties.^11^ These findings suggest that l‐carnitine supplementation could be beneficial in managing sarcopenia and frailty in patients with liver cirrhosis. Therefore, this study aims to evaluate the effects of l‐carnitine supplementation on frailty status and liver function in adults with liver cirrhosis.

## 2 METHOD

### 2.1 Study design and patients

This study is a double‐blinded, randomized, controlled clinical trial (RCT) that aimed to evaluate the effects of l‐carnitine supplementation on frailty status and liver function among adults with liver cirrhosis. This RCT was conducted from January 2022 to July 2022. The participants were selected from patients aged 18–60 years with confirmed liver cirrhosis based on pathological results, fibro scan, sonography, and clinical data, during their routine visits at the gastroenterology clinic of Abu‐Ali‐Sina Hospital, Shiraz, Iran. Patients were deemed ineligible for the study if they (1) had overt hepatic encephalopathy (defined as the presence of asterixis and disturbed level of consciousness unexplainable with other possible causes such as intracranial diseases, sepsis, hypoglycemia, electrolyte disorders, or alcohol or drug consumption); (2) had used l‐carnitine during the last consecutive year; (3) were suffering from documented hepatocellular carcinoma and uncontrolled diabetes (HbA1c > 7); and (4) had Model for End‐stage Liver Disease (MELD) scores over 20.

### 2.2 Sample size and randomization

The required study samples were estimated to be 35 patients per group based on the following formula and 95% confidence intervals (CIs), 80% test power, effect sizes of 0.70 (mean difference: 0.035, S: 0.05) allocation ratio (*r*: 1), and a 10% dropout rate.

$$n=\frac{\frac{1+r}{r}s^{2}{\left(z_{1-\alpha /2}+z_{1-\beta }\right)}^{2}}{{\left(\partial \right)}^{2}}.$$

**Figure 1:**
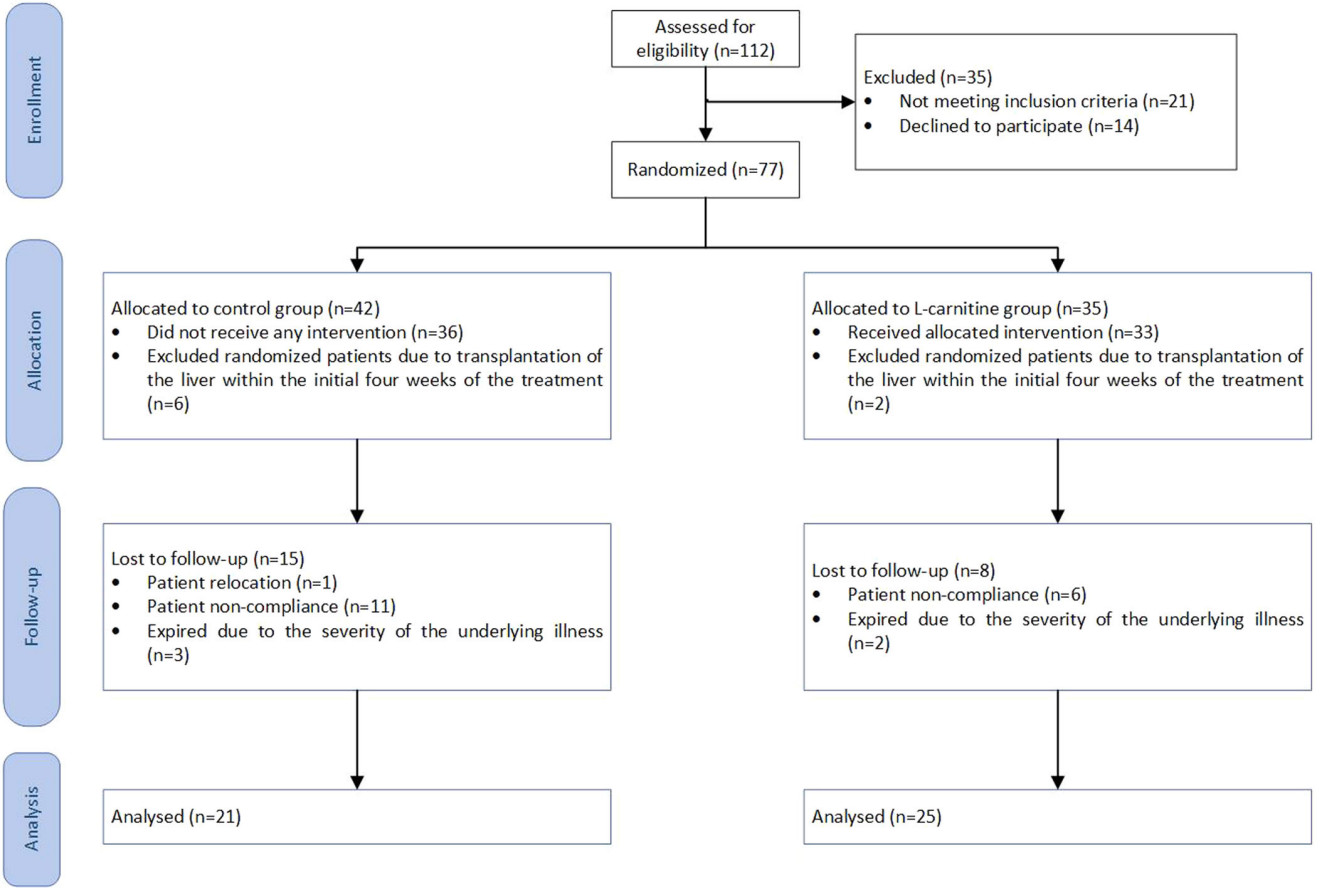
Consort flow diagram illustrating the process of participants' allocation and follow‐up through the trial.

Figure 1 shows how patients were selected and randomly allocated into the treatment and control groups. A four‐block permuted block randomization method was used. In addition, an independent third party was responsible for blindly allocating the patients to each group. The intervention types were concealed from the outcome assessors and a statistician to assess the outcomes.

### 2.3 Interventions

A total number of 77 eligible patients were randomly allocated into two arms: the intervention group received 500 mg of l‐carnitine (provided by Shahab Darman company under the license of Lonza manufacturer) orally three times a day for 2 months, along with their usual medication regimen, while the control group solely received their regular medications.

### 2.4 Data collection

Data collection was performed by medical interns who had received relative training. The patient evaluation was performed in two stages: assessing frailty status, KPSS, and anthropometric indices before starting and 3 days after completing l‐carnitine supplementation. The primary outcomes were frailty status, measured by the Karnofsky Performance Status Scale (KPSS), Freid Frailty Index (FFI), and Liver Frailty Index (LFI). The secondary outcomes included changes in renal and liver function tests. To assess frailty, we used the equation proposed by Lai et al.^12^ for LFI, whose formula is: (−0.33 * sex‐adjusted grip strength) + (−2.529 * number of squats per second) + (−0.04 * balance time) + 6, and the results were classified as frail, robust, and pre‐frail for scores ≥4.5, ≤3.2, 3.3–4.4. In accordance with Van Jacobs' scholarly investigation,^13^ an additional tool utilized to evaluate frailty is FFI, a comprehensive assessment metric that derives its score by evaluating multiple features, including weight loss, exhaustion, walk speed, activity level, and hand grip strength. A body mass index (BMI: weight/height^2^) of 25 and 30 kg/m^2^ or above was considered overweight and obese, respectively. Ordinary task performance ability was evaluated by KPSS, which ranges from 0 to 100. A higher KPSS score indicates that the patient manifests better ability in performing routine tasks and activities of daily living.^14^

The research was carried out in alignment with the ethical guidelines outlined in the Declaration of Helsinki.^15^ The Ethics Committee of Shiraz University of Medical Sciences, Shiraz, Iran, approved the study protocol (IR. SUMS. MED. REC.1400.540). Also, the study protocol was registered at the Iranian Registry of Clinical Trials (IRCT20200628047940N2 || https://irct.behdasht.gov.ir/trial/61310) on 2022.06.14. Patient's written informed consent was obtained before they were included in the study.

### 2.5 Statistical analysis

Statistical analysis was carried out and reported according to guidelines for reporting of statistics for clinical research by Assel et al., by using Statistical Package for the Social Sciences (SPSS) for Windows, Version 16.0. Chicago, SPSS Inc.^16^ Mean and standard deviation (SD) were used to report continuous variables, while frequency and percentage were used to report the rates. Normality was checked using Shapiro‐Wilk's Test. Independent t‐test (or Mann‐Whitney U test) and chi‐square test were used to compare the two groups in quantitative and qualitative variables, respectively. According to the type of data, within group analysis was performed using paired *t*‐test, Wilcoxon, Friedman, or McNemar test. For confounding factor control such as the baseline amounts of variables, we used difference‐in‐difference method analysis. All tests were performed at a two‐sided significance level of 5%.

## 3 RESULTS

### 3.1 Patients' characteristics

The enrollment and allocation processes are presented in Figure 1 using a CONSORT flow diagram. 77 eligible patients were enrolled in the study and randomly assigned to two groups, 42 were assigned to the control group and 35 to the l‐carnitine group. Eight patients discontinued their enrollment due to liver transplantation within the initial 4 weeks of the study. 23 patients (29.8%) lost to follow‐up after the initiation of the study (8 participants in the l‐carnitine and 15 in the control arm). Among the patients, 17 did not attend the follow‐up appointments due to the coronavirus disease 2019 (COVID‐19) lockdown (6 participants belonged to the intervention, while the remaining 11 belonged to the control arm). One patient in the control arm was excluded due to relocation, and five expired due to the severity of the underlying disorder (two in the l‐carnitine and three in the control arm). Consequently, a per‐protocol analysis was conducted, wherein the included patients were distributed in two groups: 21 patients in the control group and 25 patients in the l‐carnitine group. Age and MELD scores were evenly distributed across groups. The mean age of the patients was 50.5 ± 13.02 years, and 63.6% of them were male. The mean age and the mean MELD scores were 52.14 ± 10.14 years versus 49.16 ± 14.94 years, *p*: 0.325 and 15.67 ± 6.76 versus 14.77 ± 4.70, *p*: 0.533 in the intervention and control, respectively. Furthermore, there were no statistically significant disparities observed regarding the occurrence of decompensated events such as hepatic encephalopathy, ascites, jaundice, or variceal hemorrhage (*p*: 0.220), hospitalization (*p*: 0.424) and liver transplant rates (*p*: 0.513), and mortality (*p*: 0.851) between the study arms. Notably, the male gender was more prevalent in the carnitine intervention arm compared to the control arm (*p*: 0.025). Table 1 summarizes the patients' demographic and baseline clinical characteristics.

**Table 1:** Comparison of demographic and clinical characteristics between the l‐carnitine and control group.

Variables	Groups		*p* value
	Control (*n* = 42)	l‐carnitine (*n* = 35)	
Age at enrollment, mean ± SD	49.16 ± 14.94	52.14 ± 10.14	0.325
Age at diagnosis time, mean ± SD	44.95 ± 16.21	48.48 ± 11.25	0.265
Sex (male/female)	22 (52.4)	27 (77.1)	0.025
Marital (married/single)	38 (90.5)	33 (94.4)	0.535
MELD score, mean ± SD	14.77 ± 4.70	15.67 ± 6.76	0.533
Height, mean ± SD	169.26 ± 9.06	171.39 ± 9.15	0.313
Comorbidities, *n* (%)	13 (30.9)	9 (25.71)	0.478
Alcohol, *n* (%)	3 (7.1)	6 (17.1)	0.174
Substance abuse, *n* (%)	6 (14.3)	5 (14.3)	>0.999
Smoker, *n* (%)	14 (33.3)	11 (31.4)	0.859
Cirrhosis			0.355
Cryptogenic, *n* (%)	4 (9.5)	3 (8.6)	
HBV/HCV, *n* (%)	8 (19.0)	6 (17.1)	
Autoimmune liver disease, *n* (%)	14 (33.3)	9 (25.7)	
NASH, *n* (%)	10 (23.8)	13 (37.14)	
Alcoholic, *n* (%)	2 (4.75)	2 (5.7)	
Others, *n* (%)	4 (9.5)	2 (5.7)	
Fatigue, *n* (%)	35 (83.3)	25 (73.5)	0.297
Hepatomegaly, *n* (%)	4 (9.5)	4(11.4)	0.785
Splenomegaly (yes)	18 (42.9)	12 (34.3)	0.443
Decompensated events, *n* (%)	36 (85.7)	33(94.3)	0.220
Abdominal pain, *n* (%)	23 (54.8)	18 (51.4)	0.770
Edema, *n* (%)	15 (35.7)	23 (65.7)	0.009
Spider angioma, *n* (%)	3 (7.1)	1 (2.9)	0.399
Palmar erythema, *n* (%)	4 (9.5)	5 (14.3)	0.517
Itching, *n* (%)	20 (47.6)	18 (51.4)	0.739
Cirrhosis hospital admission, *n* (%)	30 (71.4)	22 (62.9)	0.424
Death, *n* (%)	2 (4.8)	2 (5.7)	0.851
Liver transplant, *n* (%)	7 (16.7)	4 (11.4)	0.513

*Note*: Decompensated events include hepatic encephalopathy, ascites, Jaundice, or variceal hemorrhage. Substance abuse defined as substance use for over 12 months. We used a *t*‐test or chi‐square test.

Abbreviations: HBV, hepatitis B virus; HCV, hepatitis C virus; MELD, model for end‐stage liver disease; NASH, nonalcoholic steatohepatitis.

### 3.2 Effects of l‐carnitine on frailty status and physical function

Table 2 outlines the assessment of frailty and anthropometric indices. There was no significant difference in the KPSS changes (0.4 ± 15.13 vs. 3.80 ± 12.44, *p*: 0.435) between the groups during the follow‐up period. Our results indicated that there was no significant difference between the l‐carnitine and the control groups regarding Δ LFI (0.00 ± 0.89 vs. 0.11 ± 0.56, *p*: 0.142) or any of its components including Δ balance, Δ grip strength, and Δ chair stands (*p* > 0.05). Furthermore, despite observing a significant reduction in walking speed in the control group (*p*: 0.020), there was a perceptible inclination towards safeguarding against the decline of speed in the l‐carnitine group (Δ walk speed: 0.04 ± 3.24 vs. 1.62 ± 2.93, *p*: 0.091). However, when it comes to other contributing factors involved in the FFI calculation, such as weight loss, exhaustion, activity level, and BMI, there was no significant distinction between the two groups (*p* > 0.05). Moreover, no significant difference was observed between the l‐carnitine and the control group regarding ΔFFI (−0.320 ± 1.46 vs. −0.350 ± 1.38, *p*: 0.945) after 8 weeks of treatment. Our study findings also indicated that the administration of l‐carnitine might result in a nonsignificant decrease in the MELD score (*p*: 0.083). Furthermore, the comparison between the control and treatment groups revealed a nonsignificant reduction in the mean changes of MELD score associated with l‐carnitine treatment. Respectively, the mean change in the MELD score was 1.94 ± 4.32 in the l‐carnitine group and 1.00 ± 4.22 in the control group (*p*: 0.073) (Table 2).

**Table 2:** Comparison of frailty assessment tools in l‐Carnitine and control groups at baseline and end of the trial.

Variables		Groups		*p* value
		Control (*n* = 21)	l‐carnitine (*n* = 25)	
Weight	Baseline	71.05 ± 17.06	79.10 ± 13.16	0.083
	Follow‐up	69.52 ± 16.26	77.40 ± 12.69	0.072
	Change	−1.52 ± 4.98	−1.70 ± 5.24	0.911
	*p* value (within‐group)	0.175	0.118	‐
BMI	Baseline	24.95 ± 5.44	27.11 ± 3.79	0.238
	Follow‐up	24.44 ± 5.12	26.52 ± 3.54	0.323
	Change	−0.51 ± 1.70	−0.58 ± 1.80	0.460
	*p* value (within‐group)	0.181	0.115	
Balance	Baseline	29.21 ± 2.54	30.0 ± 0	0.128
	Follow‐up	28.45 ± 3.90	28.8 ± 6.0	0.822
	Change	−0.75 ± 2.47	−1.20 ± 6.0	0.754
	*p* value (within‐group)	0.177	0.327	
Chair stand	Baseline	18.34 ± 6.49	15.94 ± 6.77	0.233
	Follow‐up	16.77 ± 8.45	18.71 ± 11.84	0.536
	Change	−1.56 ± 10.23	2.77 ± 9.23	0.142
	*p* value (within‐group)	0.492	0.154	‐
LFI	Baseline	4.31 ± 0.62	4.12 ± 0.58	0.233
	Follow‐up	4.42 ± 0.85	4.11 ± 1.02	0.536
	Change	0.11 ± 0.56	0.00 ± 0.89	0.142
	*p* value (within‐group)	0.413	0.972	‐
KPSS	Baseline	83.33 ± 14.94	82.00 ± 12.58	0.917
	Follow‐up	79.52 ± 16.57	82.40 ± 16.40	0.600
	Change	−3.80 ± 12.44	0.40 ± 15.13	0.435
	*p* value (within‐group)	0.176	0.896	‐
Walking time	Baseline	7.02 ± 1.61	7.28 ± 2.80	0.616
	Follow‐up	8.63 ± 3.07	7.41 ± 2.98	0.181
	Change	1.62 ± 2.93	0.04 ± 3.24	0.091
	*p* value (within‐group)	0.020	0.945	‐
Hand grip	Baseline	22.39 ± 12.01	26.85 ± 10.86	0.216
	Follow‐up	20.22 ± 13.45	27.13 ± 13.66	0.111
	Change	−2.16 ± 5.86	0.28 ± 9.12	0.327
	*p* value (within‐group)	0.136	0.880	‐
Weight loss	Baseline	30 (71.4)	24 (68.6)	0.785
	Follow‐up	14 (66.7)	11 (45.8)	0.161
	*p* value (within‐group)	0.453	0.424	
Physical endurance/energy	Baseline	22 (52.4)	17 (48.6)	0.739
	Follow‐up	9 (42.9)	12 (48.0)	0.727
	*p* value (within‐group)	0.5	>0.999	
Baseline exhaustion	Every day	9 (21.4)	7 (20.0)	0.786
	Every week	6 (14.3)	7 (20.0)	
	Once	3 (7.1)	1 (2.9)	
	Not at all	24 (57.1)	20 (57.1)	
Follow‐up exhaustion	Every day	5 (23.8)	4 (16.0)	0.880
	Every week	3 (14.3)	3 (12.0)	
	Once	1 (4.8)	2 (8.0)	
	Not at all	12 (57.1)	16 (64.0)	
	*p* value	0.954		
Baseline activity level	3 times per week	3 (7.1)	6 (17.1)	0.110
	1–2 times per week	4 (9.5)	3 (8.6)	
	1–3 times per month	0 (0)	3 (8.6)	
	Hardly ever/never	35 (8.3.3)	23 (65.7)	
Follow‐up activity level	3 times per week	7 (33.3)	6 (24.0)	0.596
	1–2 times per week	1 (4.8)	3 (12.0)	
	1–3 times per month	0(0)	1 (1.0)	
	Hardly ever/never	13 (61.9)	15 (60.0)	
	*p* value	0.112	0.287	‐
FFI	Before	2.85 ± 1.13	2.68 ± 1.31	0.650
	After	2.50 ± 1.46	2.36 ± 1.43	0.750
	Change	−0.350 ± 1.38	−0.320 ± 1.46	0.945
	*p* value (within‐group)	0.273	0.285	‐
MELD score	Baseline	14.77 ± 4.70	15.75 ± 6.76	0.519
	Follow‐up	14.69 ± 5.87	14.58 ± 6.09	0.963
	Change	1.00 ± 4.22	−1.94 ± 4.32	0.073
	*p* value	0.410	0.083	‐

*Note*: For continuous variables, we employed an independent sample *t*‐test to compare between two groups, and a paired sample *t*‐test for within‐group comparisons in each respective group. Concerning categorical variables, we utilized the chi‐square test for comparisons between two groups, and the McNemar or Friedman test for within‐group comparisons in each respective group.

Abbreviations: FFI, Freid Frailty Index; KPSS, Karnofsky Performance Status Scale; LFI, Liver Frailty Index; MELD, model for end‐stage liver disease.

### 3.3 Effects of l‐carnitine on laboratory measurements

Table 3 presents the laboratory parameters gathered in the course of clinical care. L‐carnitine did not affect renal function (blood urea nitrogen or creatinine), platelet count, hemoglobin concentration, sodium, and potassium levels between the two randomized groups (*p* > 0.05). At the end of week 8, a significant reduction from baseline in alanine aminotransferase (ALT) was observed in the l‐carnitine group (*p*: 0.043), while no significant change was observed in the control group. At week 8, the l‐carnitine group showed a mean change in ALT level from baseline of −13.47 ± 29.28 IU/L (*p*: 0.043), while the control group had an insignificant mean change of −6.24 ± 19.32 IU/L (*p*: 0.201) (Table 3). Furthermore, our findings indicated that total serum bilirubin showed a nonsignificant trend toward decreasing in the l‐carnitine group (*p*: 0.064). Nonetheless, no notable alterations were observed in aspartate aminotransferase, alkaline phosphatase, direct serum bilirubin, total protein, and albumin levels between the two randomized groups (*p* > 0.05).

**Table 3:** Comparison of laboratory variables in l‐carnitine and control groups at baseline and end of the trial.

Laboratory findings		Groups		*p* value
		Control (*n* = 21)	l‐carnitine (*n* = 25)	
**BUN (mg/dL)**	Baseline	16.55 ± 7.23	16.64 ± 7.76	0.930
	Follow‐up	17.06 ± 10.03	16.6 ± 6.46	0.707
	Change	0.41 ± 10.11	−0.40 ± 6.02	0.773
	*p* value (within‐group)	0.869	0.782	‐
**Creatinine (mg/dL)**	Baseline	0.99 ± 0.19	1.02 ± 0.37	0.139
	Follow‐up	1.05 ± 0.44	1.03 ± 0.23	0.893
	Change	0.05 ± 0.40	−0.00 ± 0.31	0.694
	*p* value (within‐group)	0.577	0.906	‐
**AST (IU/L)**	Baseline	56.25 ± 31.80	60.61 ± 36.74	0.996
	Follow‐up	49.0 ± 26.42	49.04 ± 33.73	0.996
	Change	−7.25 ± 28.63	−11.46 ± 40.40	0.711
	*p* value (within‐group)	0.312	0.194	‐
**ALT (IU/L)**	Baseline	38.83 ± 27.31	44.38 ± 29.34	0.236
	Follow‐up	32.58 ± 21.10	30.90 ± 21.97	0.811
	Change	−6.24 ± 19.32	−13.47 ± 29.28	0.385
	*p* value (within‐group)	0.201	0.043	‐
**ALP (IU/L)**	Baseline	292.22 ± 164.66	319.45 ± 195.59	0.611
	Follow‐up	267.18 ± 142.42	261.86 ± 156.59	0.802
	Change	−25.04 ± 166.97	57.59 ± 200.31	0.600
	*p* value (within‐group)	0.558	0.192	‐
**Total serum bilirubin (mg/dL)**	Baseline	2.54 ± 3.13	2.79 ± 1.92	0.289
	Follow‐up	3.22 ± 5.36	2.18 ± 1.41	0.402
	Change	0.67 ± 6.12	−0.6 ± 1.41	0.359
	*p* value (within‐group)	0.655	0.064	‐
**Direct serum bilirubin (mg/dL)**	Baseline	1.17 ± 1.76	1.01 ± 0.92	0.184
	Follow‐up	1.81 ± 4.29	0.76 ± 0.73	0.278
	Change	0.63 ± 4.78	0.24 ± 1.03	0.418
	*p* value (within‐group)	0.602	0.300	‐
**Total protein (g/dL)**	Baseline	7.34 ± 0.28	7.05 ± 1.32	0.896
	Follow‐up	7.30 ± 0.53	6.67 ± 0.71	0.057
	Change	−0.04 ± 0.47	−0.37 ± 1.72	0.684
	*p* value (within‐group)	0.859	0.558	‐
**Albumin (g/dL)**	Baseline	3.79 ± 0.69	3.53 ± 0.55	0.683
	Follow‐up	3.66 ± 0.81	3.52 ± 0.54	0.663
	Change	−0.12 ± 0.36	−0.00 ± 0.71	0.631
	*p* value (within‐group)	0.269	0.969	‐
**PT (seconds)**	Baseline	16.54 ± 4.50	18.58 ± 5.22	0.288
	Follow‐up	19.05 ± 6.55	16.66 ± 3.54	0.178
	Change	2.51 ± 4.13	−1.92 ± 4.90	0.008
	*p* value (within‐group)	0.040	0.096	‐
**PTT (seconds)**	Baseline	33.97 ± 9.58	35.40 ± 5.05	0.223
	Follow‐up	33.72 ± 6.60	34.10 ± 5.90	0.989
	Change	−0.25 ± 8.77	−1.30 ± 4.07	0.726
	*p* value (within‐group)	0.933	0.036	‐
**INR**	Baseline	1.44 ± 0.4	1.57 ± 0.53	0.213
	Follow‐up	1.55 ± 0.49	1.42 ± 0.32	0.324
	Change	0.11 ± 0.18	−0.15 ± 0.39	0.024
	*p* value (within‐group)	0.036	0.312	‐
**WBC (×10** ^**9**^ **/L)**	Baseline	4.75 ± 1.84	6.64 ± 3.09	0.954
	Follow‐up	4.79 ± 1.94	6.03 ± 2.49	0.097
	Change	0.037 ± 1.95	−0.61 ± 1.65	0.264
	*p* value (within‐group)	0.939	0.096	‐
**Platelet (×10** ^**9**^ **/L)**	Baseline	131.87 ± 81.61	154.57 ± 141.74	0.984
	Follow‐up	150.62 ± 140.22	175.28 ± 165.35	0.933
	Change	18.75 ± 160.30	−20.66 ± 0.98	0.362
	*p* value (within‐group)	0.647	0.348	‐
**Hb (g/dL)**	Baseline	12.47 ± 2.26	12.67 ± 2.20	0.623
	Follow‐up	12.72 ± 2.05	12.69 ± 2.59	0.949
	Change	−0.25 ± 1.32	−0.019 ± 1.84	0.655
	*p* value (within‐group)	0.432	0.963	‐
**Na (mEq/L)**	Baseline	138.15 ± 4.07	135.46 ± 5.13	0.081
	Follow‐up	137.69 ± 3.30	133.16 ± 22.54	0.452
	Change	−0.46 ± 4.84	−2.30 ± 22.68	0.777
	*p* value (within‐group)	0.737	0.672	
**K (mEq/L)**	Baseline	4.06 ± 0.77	4.34 ± 0.49	0.084
	Follow‐up	4.20 ± 0.49	4.31 ± 0.41	0.846
	Change	0.13 ± 0.87	−0.038 ± 0.64	0.538
	*p* value (within‐group)	0.572	0.817	‐

*Note*: We employed an independent sample *t*‐test to compare the two groups and a paired sample *t*‐test to examine within‐group comparisons in each respective group.

Abbreviations: ALP, alkaline phosphatase; ALT, alanine transaminase; AST, aspartate aminotransferase; BUN, blood urea nitrogen; Hb, hemoglobin; INR, international normalized ratio; K, potassium; Na, sodium; PT, prothrombin time; PTT, partial thromboplastin time; WBC, white blood cell.

Participants treated with l‐carnitine showed a decrease across all coagulation evaluations. In particular, the treatment group's prothrombin time (PT) was significantly reduced compared to the control group (*p*: 0.008). The l‐carnitine group showed a mean PT change from baseline of −1.92 ± 4.90 (*p*: 0.096), while the control group had a mean change of 2.51 ± 4.13 (*p*: 0.040). The l‐carnitine group showed a significant decrease in INR compared to the control group (*p*: 0.024). In the l‐carnitine group, the mean change in INR from baseline was −0.15 ± 0.39 (*p*: 0.312), and in the control group, it was 0.11 ± 0.18 (*p*: 0.036). Moreover, l‐carnitine decreased the partial thromboplastin time (PTT) level. This was evidenced by the mean changes in the PTT levels from baseline at week 8, which were ascertained to be −1.30 ± 4.07 (*p*: 0.036) in the l‐carnitine group and −0.25 ± 8.77 (*p*: 0.933) in the control group Table 3.

## 4 DISCUSSION

The effectiveness of timely nutritional assessment and intervention of patients with chronic liver disease has been established previously. These strategies offer significant potential in mitigating complications associated with the condition.^17^ In recent years, carnitine supplementation has attracted the attention of medical experts. Recent studies have demonstrated that l‐carnitine can improve the serum level of liver enzymes in patients with chronic liver disease.^18^, ^19^ The current study is the first to investigate the effect of l‐carnitine supplementation on frailty status and physical function in patients with liver cirrhosis. Our results suggest that l‐carnitine supplementation, despite its hepatoprotective properties, might be ineffective in short‐term frailty improvement in these patients.

The findings of our study indicated a significant reduction in ALT, PT, INR, and PTT levels following l‐carnitine supplementation. These results align with previous investigations,^18^, ^20^, ^21^ indicating the hepatoprotective effects of l‐carnitine supplementation. l‐carnitine potentially influences various coagulation parameters by mitigating oxidative stress‐induced damage. In detail, l‐carnitine can counteract oxidative damage by improving endothelial function, reducing the risk of thrombosis, and improving coagulation markers.^22^, ^23^

Although the precise mechanisms underlying the ability of l‐carnitine to reduce ALT levels in chronic liver disease are not fully understood, existing evidence indicates that l‐carnitine may improve mitochondrial function and mitigate oxidative stress within hepatocytes.^23^, ^24^, ^25^ By improving mitochondrial function, carnitine may enhance the ability of the hepatocytes to process and eliminate toxic substances from the body, thereby reducing liver damage and inflammation.^18^, ^24^

Frailty is a complex clinical condition that involves multiple systems and is associated with increased morbidity and mortality, especially in patients with liver cirrhosis.^26^ Liver cirrhosis causes frailty through different mechanisms, such as protein metabolism alteration, increasing oxidative stress, and inflammation.^6^ The regulatory effect of l‐carnitine on these factors was studied in previous research, which led to the hypothesis that it can also improve frailty.^11^

However, the results of our study suggest that short‐term l‐carnitine supplementation may not be effective in improving frailty outcomes in patients with liver cirrhosis. There were no statistically significant differences between the control and l‐carnitine groups regarding frailty parameters and physical function. Contrary to our results, Yano et al. demonstrated that l‐carnitine supplementation effectively enhanced physical activity among hemodialysis patients.^27^ Additionally, previous studies have shown that l‐carnitine treatment can have favorable effects on the functional status and frailty among prefrail older adults, survival in patients with cirrhosis, muscle cramping, and hemoglobin levels in dialysis patients, particularly patients on chronic dialysis.^28^, ^29^, ^30^ However, a clinical trial reported that although l‐carnitine was safe, it was ineffective in improving the quality of life and liver function of patients with minimal encephalopathy.^31^ Our results can be explained by the intricate nature of frailty, indicating that a solitary intervention such as l‐carnitine supplementation may not improve frailty outcomes. On the other hand, the effect of l‐carnitine supplementation on frailty outcomes may be influenced by other factors, including comorbidities, concomitant medications, and lifestyle. Our study did not meticulously control these potentially confounding variables, which may have confounded the results.

The current study did not identify significant changes in serum creatinine levels or plasma urea, indicating that short‐term l‐carnitine supplementation may not enhance renal function in patients with liver cirrhosis. In contrast, a randomized clinical trial involving 54 critically ill patients in intensive care, who received 3 g of l‐carnitine daily for 1 week, reported a significant reduction in serum creatinine levels.^20^ Additionally, Jennaro et al. found that high‐dose (≥6 g) intravenous l‐carnitine improved kidney function in septic shock patients.^32^ Our findings may be attributed to the normal baseline levels of blood urea nitrogen and creatinine in our patients, and the lower dosage of carnitine administered compared to the aforementioned studies. We recommend further investigation into the effects of l‐carnitine on renal function in future research.

This study is limited in small sample size, which may have limited our ability to detect significant differences between the groups. Secondly, the duration of the intervention was relatively short, and studies with longer durations of intervention and follow‐up may yield different results. Thirdly, the serum levels of carnitine were not measured in this study, making suboptimal serum levels possible. It is, therefore, recommended that future studies should consider measuring carnitine serum levels to determine the most efficacious doses of l‐carnitine for affected patients. Further studies with larger sample sizes and more extended treatment/follow‐up periods are also recommended. Nevertheless, our findings underscore l‐carnitine as a short‐term therapeutic option for patients suffering from liver cirrhosis. Further investigations are needed to evaluate the long‐term results, underlying mechanisms, and clinical applications.

## 5 CONCLUSION

In conclusion, l‐carnitine treatment had hepatoprotective effects associated with reduced ALT levels and may improve coagulopathy in patients with liver cirrhosis.

## AUTHOR CONTRIBUTIONS

**Nasrin Motazedian:** Conceptualization; methodology; project administration; supervision; writing—review and editing. **Anita Ashari:** Investigation; validation; data curation. **Niloofar Dehdari Ebrahimi:** Writing—original draft; methodology; writing—review and editing; visualization. **Mehrab Sayadi:** Software; formal analysis. **Sarina Pourjafar:** Investigation. **Nazanin Motazedian:** Investigation. **Vahid Khademi:** Investigation. **Alireza Shamsaeefar:** Investigation; validation. **Ahad Eshraghian:** Conceptualization; methodology; supervision; investigation; writing—review and editing.

## CONFLICT OF INTEREST STATEMENT

The authors declare that they have no conflicts of interest.

## ETHICS STATEMENT

Patients' written informed consent was obtained before they were included in the study, and the Ethics Committee of Shiraz University of Medical Sciences, Shiraz, Iran, approved the study protocol.

## TRANSPARENCY STATEMENT

The lead author Nasrin Motazedian affirms that this manuscript is an honest, accurate, and transparent account of the study being reported; that no important aspects of the study have been omitted; and that any discrepancies from the study as planned (and, if relevant, registered) have been explained.

## Data Availability

The data that support the findings of this study are available from the corresponding author upon reasonable request. All authors have read and approved the final version of the manuscript. Niloofar Dehdari Ebrahimi and Anita Ashari have full access to all of the data in this study and take complete responsibility for the integrity of the data and the accuracy of the data analysis.
